# Digital affordances: how entrepreneurs access support in online communities during the COVID-19 pandemic

**DOI:** 10.1007/s11187-021-00540-2

**Published:** 2021-10-11

**Authors:** Marie Madeleine Meurer, Matthias Waldkirch, Peter Kalum Schou, Eliane Léontine Bucher, Katrin Burmeister-Lamp

**Affiliations:** 1 Entrepreneurship & Family Firm Institute (EFFI), Department of Entrepreneurship and Innovation, EBS University of Business and Law, Gustav-Stresemann-Ring 3, 65189 Wiesbaden, Germany; 2 Department of Strategy and Entrepreneurship, BI Norwegian Business School, Nydalsveien 37, 0484 Oslo, Norway; 3 Department of Communication and Culture, BI Norwegian Business School, Nydalsveien 37, 0484 Oslo, Norway

**Keywords:** Entrepreneurial support, Online communities, Affordances, COVID-19, Big data, L26, M10, M13

## Abstract

COVID-19 has caused significant and unforeseen problems for entrepreneurs. While entrepreneurs would normally seek social support to help deal with these issues, due to social distancing, physical networks are often not available. Consequently, entrepreneurs must turn to alternative support sources, such as online communities, raising the question of how support is created in such spaces. Drawing on an affordance perspective, we investigate how entrepreneurs interact with online communities and base our qualitative analysis on conversation data (76,365 posts) from an online community of entrepreneurs on Reddit during the COVID-19 pandemic. Our findings draw out four affordances that online communities offer to entrepreneurs (resolving problems, reframing problems, reflecting on situations, refocusing thinking and efforts), resulting in a framework of entrepreneurial support creation in online communities. Thus, our study contributes to debates around (1) entrepreneurs’ support during COVID-19 and (2) digital affordances in the entrepreneurship context.

## Abstract (plain language summary)

As an entrepreneur (during the COVID-19 pandemic), start looking for support in online communities—they are more than just knowledge repositories! While access to support is severely hampered by social distancing measures during the COVID-19 pandemic, entrepreneurs can still access support in online communities. Yet, depending on how entrepreneurs approach them, communities offer more than just simple advice: depending on how entrepreneurs frame their posts and interact with the community, they can manage what support they receive from online communities. (1) Online communities can help resolve problems and collect critical resources in times of crisis, such as digital marketing tools to compensate client loss. (2) Online communities can support entrepreneurs to waterproof ideas in early venture stages through feasibility checks so that entrepreneurs can better evaluate opportunities. (3) Online communities can help understand and reflect on new emerging topics, such as work from home. (4) Online communities can provide tailor-made plans for entrepreneurs by engaging in frequent interaction with support seekers. Taken together, online communities can assist entrepreneurs in their actions which are especially important in times of great uncertainty.

## Introduction

Social networks are a crucial success factor for entrepreneurs as they provide them with essential support, such as resources for their businesses, information, and emotional aid (e.g., Gloor et al., 2018; Jack, 2005; Klyver et al., 2018). However, during the COVID-19 crisis, entrepreneurs’ ability to seek support in their network is severely hampered by governmental restrictions, namely social distancing measures, that aim to contain the outbreak of the pandemic (c.f. Giones et al., 2020; Kuckertz et al., 2020). Social distancing measures mean that entrepreneurs cannot network or meet up in physical settings with peers or professional advisors and mentors, which are crucial for entrepreneurs to access social support (Kuhn & Galloway, 2015; Kuhn et al., 2017; Vissa & Bhagavatula, 2012). While these traditional offline spaces might be closed off due to COVID-19 (Majchrzak & Shepherd, 2021), digital spaces are open for entrepreneurs to engage in. Research points especially to online communities as alternative sources of critical social support (Giones et al., 2020; Majchrzak & Shepherd, 2021). In the entrepreneurship context, online communities refer to digital spaces where geographically dispersed, entrepreneurial-minded individuals come together and support each other (Kuhn et al., 2016, 2017) by exchanging knowledge and providing resources or emotional aid (e.g., Faraj et al., 2016; Kraut & Resnick, 2012; Kuhn et al., 2016). Even outside the COVID-19 pandemic, researchers find that entrepreneurs can obtain support more efficiently in online communities in comparison to other sources (Kuhn et al., 2017). As such, recent work on digital entrepreneurship explicitly highlights online communities as a key “digital infrastructure” for entrepreneurs (Nambisan, 2017, p. 1032).

Even though researchers, ranging from entrepreneurship to information systems, are unpacking how actors may benefit from seeking support in online communities (Faraj et al., 2016; Leonardi, 2014, 2018; Nambisan, 2017; Treem & Leonardi, 2013), surprisingly, little is known about how this support is created by online communities (Faraj et al., 2016). While recent research in entrepreneurship investigates the impact of membership in online communities and networks, such as LinkedIn (Gloor et al., 2018; Song et al., 2019), there is less attention on how entrepreneurs interact in online communities. Consequently, while we have an increasing knowledge about how actors in organizations use online communities for support (Leonardi, 2017), there is a lack of understanding about how entrepreneurs access support from online communities by interacting with the community. To better understand this, we follow Nambisan’s call (2017, p. 1043) to draw on the theoretical perspective of affordances, which are “action possibilities and opportunities that emerge from actors engaging with focal technology” (Faraj & Azad, 2012, p. 238). This perspective is fitting as it is widely understood that the affordances, which emerge when actors engage with technology, such as online communities, heavily depend on interactions, i.e., technology-user transactions, and the specific context (Faraj & Azad, 2012; Leonardi, 2012; Treem & Leonardi, 2013). In other words, the theory of affordances explains how user interactions shape what a focal technology might offer (Faraj & Azad, 2012). For example, Reddit’s online communities allow users to discuss news, share tips on baking, or arrange political activity (Jost et al., 2018), depending on (1) how users perceive the online community technology (e.g., as a space for exchanging news, for finding recipes, or for mobilizing political support), and (2) how they engage with the community technology (e.g., as a space to ask for support, to give advice, or to inform and motivate others) (c.f. Nambisan, 2017). Thus, which support users can receive from online communities during COVID-19 depends on their interactions with the community, which then produces certain affordances that can help entrepreneurs. Following this theoretical perspective, we conceptualize entrepreneurs’ online communities as malleable contexts for support that emerge through the interaction between entrepreneurs and the online community (Faraj & Azad, 2012; Leonardi, 2012; Leonardi & Treem, 2012). Therefore, we ask *how do different support affordances for entrepreneurs emerge from an online community during COVID-19 and what form do they take?*

To answer this question, we investigate conversation data from a large online community of entrepreneurs during the COVID-19 crisis. Specifically, we collected all conversations from the online community r/startups on Reddit between January and July 2020, which resulted in a total of 76,365 posts. To identify relevant conversations surrounding COVID-19 in this initial dataset, we applied a self-developed COVID-19 dictionary. Following this sampling strategy, we ended up with a total of 3903 relevant posts, which we then qualitatively analyzed in line with recognized methods for social media data (c.f. Bucher et al., 2021; Mckenna et al., 2017).

Our findings outline that depending on how entrepreneurs frame initial posts and engage with the community, four affordances emerge in online communities: (1) online communities can *resolve* problems by providing simple solutions that completely meet the addressed support seeker’s need. (2) Online communities can *reframe* more complex problems through simplification and challenging of initial submissions. (3) Online communities can make entrepreneurs *reflect* on economic, personal, or business situations through deeper discussions. (4) Online communities can *refocus* efforts and thinking of entrepreneurs by shaping future actions and orientations within conversations.

Taken together, our findings make two theoretical contributions. *First*, we contribute to the debate around where and how entrepreneurs can access support during crises (Giones et al., 2020; Kuckertz et al., 2020). We outline online communities as spaces where entrepreneurs can access support that can facilitate entrepreneurial action during crises (Shepherd, 2020) and may thus help entrepreneurs to reduce uncertainty and create future entrepreneurial action (Giones et al., 2020; McMullen & Shepherd, 2006). *Second*, we add insight to the intersection between entrepreneurship and the research on affordance theory (Faraj & Azad, 2012; Faraj et al., 2016; Leonardi, 2018; Nambisan, 2017) by drawing out four unique affordances for support-seeking entrepreneurs (*resolving, reframing, reflecting, and refocusing*)*.* So doing, we extend current thinking about how online communities can provide different types of resources to entrepreneurs (Nambisan, 2017). Further, our study enriches and complements the emergent debate on the role of digital spaces for entrepreneurs (Obschonka & Audretsch, 2020) by investigating entrepreneurs’ interactions with online communities.

## Theoretical background

### COVID-19, social support, and the role of online communities for entrepreneurs

The COVID-19 pandemic has a disproportionate impact on entrepreneurs, as research indicates that especially entrepreneurs are hit hard financially due to lockdowns (Block et al., 2021; Cowling et al., 2020). More precisely, during COVID-19, it is far more probable that entrepreneurs suffer higher income losses than employees (Graeber et al., 2021). Beyond the financial impact, research indicates that entrepreneurs may also suffer emotionally (Giones et al., 2020) since oftentimes their ventures are at risk. Furthermore, governmental measures and restrictions put an additional toll on entrepreneurs’ well-being, increasing for instance their feeling of loneliness (e.g., Kniffin et al., 2020; Williamson et al., 2021).

As an essential proactive strategy to cope with these emerging challenges during the COVID-19 pandemic, entrepreneurs rely on social support that equips them with crucial resources from their social environment (c.f. Kim et al., 2013; Tremblay & Simard, 2018). For instance, social support theory suggests that social support can reduce stress through uncertainty reduction and theoretical problem-solving (Fielden & Hunt, 2011; Huang et al., 2019; Pfeil, 2009), restore emotional stability by providing “love, sympathy, and encouragement” (Huang et al., 2019, p. 398), and can buffer negativity and enhance positivity (e.g., Barak et al., 2008; Baron, 2008; Bavik et al., 2020; Rodgers & Chen, 2005; Wiklund et al., 2019). Therefore, research highlights that entrepreneurs rely on social support to reduce stress during a crisis (Giones et al., 2020; Klyver et al., 2020).

However, due to lockdowns and social distancing, entrepreneurs may no longer be able to use their existing network or actively network to find necessary support (Giones et al., 2020; Kuckertz et al., 2020). Instead of traditional, offline social networks, recent research indicates that online communities can serve as alternative sources where entrepreneurs can access forms of social support (Giones et al., 2020; Kuhn & Galloway, 2015; Kuhn et al., 2017). Online communities can, in parts, be even more efficient in providing support than traditional networks (Kuhn et al., 2017) due to their specific characteristics.

*First*, online communities connect support seekers with like-minded others beyond their existing and often local network (Faraj et al., 2016). Thus, online communities can provide novel support beyond an entrepreneurs’ traditional network (Hajli, 2018; Kuhn et al., 2016). *Second*, online communities are often anonymous, meaning there are usually no established relationships between support seekers and responding community members. While anonymous support may not be as trustworthy (Kuhn et al., 2016; Łobacz et al., 2016), anonymity also has clear benefits. In particular, loose community bonds enable entrepreneurs to freely and safely disclose controversial and potentially shameful issues, such as failure or mental health problems (c.f. Huang et al., 2019). Consequently, entrepreneurs can seek support without fearing personal judgment or being blamed for specific questions (c.f. Turner, 2001). *Third*, when turning to online communities, entrepreneurs can access support without temporal and spatial boundaries (Hwang et al., 2015; Kuhn et al., 2016). *Fourth*, online communities enable new ways of entrepreneurial learning “through passive observation but also through active, discursive interactions” (Schou et al., 2021, p. 3). *Last*, online communities can provide tacit knowledge flows, entailing that such spaces “allow participants to share hard-to-codify knowledge such as competence and experience” (Faraj et al., 2016, p. 669). In this regard, scholars show that knowledge from online communities can drive opportunity recognition and realization (Autio et al., 2013), which can further have a positive impact on business growth (Kuhn et al., 2016).

While there is a broad understanding of online communities as supportive environments, Faraj and colleagues (Faraj et al., 2016) already indicate that support exchange is highly complex since it involves community dynamics. What online communities offer to entrepreneurs is, thus, not inherently static. For example, the community will react differently depending on whether entrepreneurs desire to use the community to test out a new opportunity (Autio et al., 2013), or whether they are seeking to deal with failure (c.f. Fisch & Block, 2021). This phenomenon where a community may shift its characteristics and offer different responses depending on the inquiry is known to information systems research as affordances (Leonardi, 2011; Leonardi & Treem, 2012), a theoretical perspective that enables us to understand online interactions.

A common difficulty in investigating “online interactions is the conflation between the enabling technologies known as social media and (…) online communities” (Faraj et al., 2016, p. 671). While social media channels, such as social networks, microblogs, or wikis, are designed to facilitate social connectivity (e.g., Faraj et al., 2016; Majchrzak et al., 2013; Treem & Leonardi, 2013), online communities do not focus on individual networks as they are usually anonymous (e.g., Kuhn et al., 2016; Łobacz et al., 2016). Therefore, social connections are formed differently. For example, it is not possible to “befriend” someone like on Facebook. However, this also means that the growing body of research in entrepreneurship, investigating the role of social media for entrepreneurs (e.g., Fisch & Block, 2021; Obschonka et al., 2017), can only give us a vague idea of how entrepreneurs interact with online communities.

### Understanding support in online communities through an affordance perspective

In understanding online community interactions, we, therefore, draw on the theoretical perspective of affordances that has received increased interest in information systems and organization as well as management theory (Leonardi & Vaast, 2017). Building on affordances as action possibilities and opportunities that emerge when a social agency (user, here, support-seeking entrepreneur) interacts with a material agency (technology, here, online communities) (e.g., Faraj & Azad, 2012; Leonardi, 2011, 2017; Nambisan et al., 2019), we argue that online communities do not provide the same inherent set of features to every user but what online communities offer depends on the user’s intent and approach. Using an affordance perspective, research in information systems has unpacked how actors may use technology to solve problems of various kinds. For example, Leonardi (2018) shows how the emerging affordances of organizational online communities allow employees to build up shared knowledge and understanding of each other. As entrepreneurship scholars have become more interested in digital aspects of entrepreneurship (Autio et al., 2018; Nambisan, 2017; Nambisan et al., 2019), they have argued for improving the understanding of how affordances emerge when entrepreneurs engage with digital technology. Nambisan (2017, p. 1043), for instance, argues that affordances “could prove invaluable in understanding the varying interpretations of the same stimuli by different entrepreneurs […] and thereby, on their future actions and outcomes.”

While there is an impetus to better understand the affordances that entrepreneurs have access to both in regular times and especially in times of crisis (Majchrzak & Shepherd, 2021), several important gaps remain. *First*, while we know online communities can be spaces for support for entrepreneurs (Kuhn et al., 2017), we lack insights into how entrepreneurs may access this support through engaging in the online communities. In contrast to social networks, a focus on affordances goes beyond the presence or position in interactions, instead of focusing on interaction and the resulting action possibilities (Leonardi, 2012). Furthermore, while there have been recent calls for entrepreneurship researchers to engage with the affordance perspective (Autio et al., 2018; Nambisan, 2017; Nambisan et al., 2019), entrepreneurship research has not yet done so in a significant way (Smith et al., 2017). Autio et al. (2018) therefore, plea for closing this gap and argue that affordances offered by online communities serve critical functions. For example, they may be key pieces in building digital ecosystems. Given the major impact of COVID-19, it is plausible that new affordances of online communities are unpacked as entrepreneurs seek to overcome business problems and lack of physical social networks incurred by the COVID-19 crisis.

In this paper, we, therefore, seek to extend recent theorizing in the entrepreneurship literature that has sought to unpack how digital entrepreneurship affordances emerge (Autio et al., 2018; Nambisan, 2017; Nambisan et al., 2019). Furthermore, we seek to add evidence to the recent proposals that online community affordances are key to entrepreneurs during the COVID-19 crisis (Majchrzak & Shepherd, 2021; Shepherd, 2020). To do so, we study a large community of entrepreneurs on Reddit during the COVID-19 pandemic.

## Methodology

This study is based on a large set of submissions and comments from an online community of entrepreneurs during the COVID-19 pandemic. In line with other studies that investigate big datasets in the entrepreneurship literature (e.g., Bloh et al., 2020; Fisch & Block, 2021; Obschonka & Fisch, 2018; Obschonka et al., 2017, 2020; Prüfer & Prüfer, 2020), we used a stepwise process to access information within the vast amount of posts, which helps us answer our research question. More precisely, we investigate community conversations in four steps (Table 1)*.* (1) We used a self-developed Python script to collect all conversations (submissions and comments) over 7 months from the online community. (2) We developed a script in R to filter data and to identify relevant discussions related to COVID-19 through a dictionary. (3) Within these discussions, we identified and coded different characteristics of support-seeking during the COVID-19 pandemic, community response patterns, and affordances combining the support seeker and the community side. (4) Drawing on our analysis, we then built a model that shows how affordances in online communities are created through community interactions. Thereby, we identified how conversations develop and how support seekers engage with the community to receive the help they need. In particular, we were observing whether discussions emerge and if as well as how support seekers return to the community.

**Table 1:**
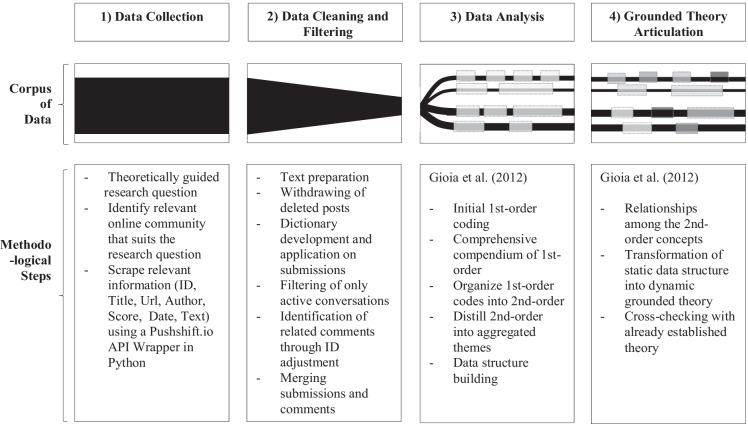
Methodology in four steps (own depiction based on Bucher et al. (2021)).

### Data collection

To identify and analyze the social support-seeking behavior of entrepreneurs in online communities during COVID-19, we collected conversation data from an online community of entrepreneurs on Reddit (r/startups). The online community connects entrepreneurs anonymously and fosters discussions “around startups, not traditional businesses” (r/startups, 2020) according to the community guidelines. Our primary reason for selecting this forum was its purpose to “support others, educate others, inspire others, and foster authentic relationships” (r/startups, 2020). Submissions that do not meet this purpose and are not “kind and supportive” are withdrawn by moderators (r/startups, 2020). Furthermore, all posts are anonymous and moderators highly encourage community members to provide as many details as possible to receive the support they need.

As of July 2020, the online community counted over 460,000 members from various countries, with about 300 to 1000 members being online at any given time. Due to missing geographical and timely constraints of online communities (e.g., Hwang et al., 2015; Kuhn et al., 2016), conversations are not bound to a national context. During the pandemic, the community gained even more relevance counting about 800,000 members in August 2021. With the help of a self-developed script, we collected all submissions and comments between January 1 and July 7, 2020 using the Reddit API Wrapper PRAW, thus capturing the first COVID-19-related lockdowns and their impact on entrepreneurs. The collected data encompasses a total of 76,365 community posts, of which 11,487 are submissions and 64,878 are comments. While submissions have an average of 5.6 comments, the number of comments varies significantly with some submissions garnering dozens of comments and others receiving very few or none at all.

### Data filtering

To access COVID-19-related conversations, we used a stepwise data filtering process (Appendix 1). In particular, we prepared the text for filtering and removed deleted posts which resulted in a sample of 63,160 posts. Then, we inductively developed a “COVID-19 dictionary” to identify the relevant conversations within the vast amount of posts (Humphreys & Wang, 2018). In particular, we were first looking for conversations that contained the words *corona*, *covid*, *lockdown*, *pandemic*, and *virus*. Out of 63,160 non-deleted posts, 1156 posts contained at least one of the relevant keywords. We then read 300 relevant posts to identify further words and phrases that connect to COVID-19. All in all, besides the five previous words, we found twenty further words that relate to the COVID-19 crisis (Appendix 2). Words were generally reduced to their stem to include variations of the same term. For instance “isolation,” “isolated,” and “isolating” were covered by their stem isolat*. After checking the additional terms for quantity and false positives, we added the following words to our dictionary, which are also nearly exclusively related to COVID-19: *quarantine*, and *work from home.* Applying this dictionary to the dataset, we identified 409 submissions that contained at least one of the relevant terms directly relating to the COVID-19 pandemic. Of these submissions, we selected all active conversations with more than five comments, resulting in 172 submissions and 3731 related comments (3903 posts in total) which encompass around 6% of non-deleted posts between January and July 2020 in the r/startups community.

### Data analysis

We followed a stepwise coding process to gain insight into the affordances that emerge when entrepreneurs seek support in online communities during the COVID-19 pandemic. We first identified and labeled specific author types to quickly understand who is talking in the community. The following types are typical for Reddit data: (a) initial support seeker posts, (b) community comments, (c) support seeker comments, (d) moderator comments, (e) bot comments. Then, we removed moderator and bot comments because these posts can be classified as noise within the conversations. Finally, we followed the coding process described by Gioia et al. (2012), analyzing entire conversations instead of individual posts to better map interactions. In line with best qualitative practice, we coded the submissions first individually and later compared our codes (Pratt et al., 2020).

**Fig. 1:**
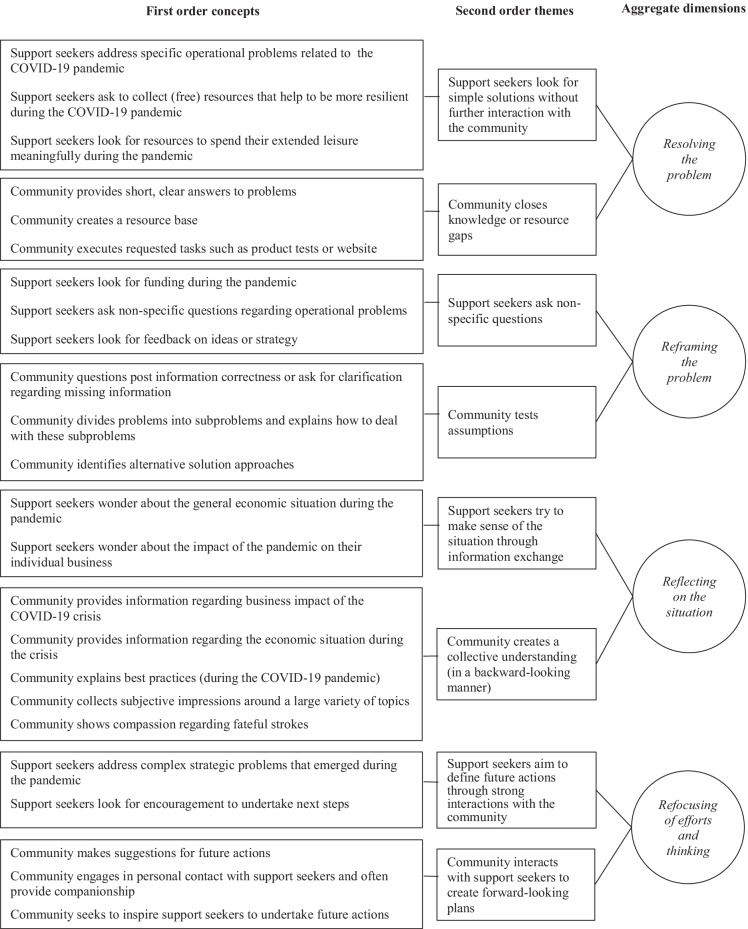
Data structure on affordances in online communities

First, we coded information terms that remain close to the text which naturally resulted in a large number of codes (Gioia et al., 2012) that refer to both support seeker and community behavior. For instance, we found that the community often asked for further clarification to better understand the nature and extent of the support seeker’s issue. Second, we identified similarities and differences of the preliminary codes (c.f. Strauss & Corbin, 1998) and specified the deeper structure of the data which reduced the number of categories (Gioia et al., 2012). Here, we specified how the identified themes suggest concepts that help us to understand which action possibilities emerge when support-seeking entrepreneurs engage with online communities (c.f. Gioia, 2004). For instance, our findings indicate the “community tests assumptions” through asking for clarification, dividing problems into subproblems, and identifying alternative approaches. Third, we distilled the 2nd-order themes into aggregate dimensions (c.f. Gioia, 2004), i.e., emerging affordances. For example, we show how support seekers asking nonspecific questions and the community challenging their assumptions unfolds in an affordance of “reframing the problem.” The results of our analysis are compiled in a data structure (Fig. 1), which provides “a graphic representation of how we progressed from raw data to terms and themes in conducting the analyses” (Gioia et al., 2012, p. 20).

### Grounded theory articulation

In the last step, we transformed the data structure, i.e., the static picture of community interactions during the COVID-19 pandemic, into a grounded theory model in line with established qualitative research methods (c.f. Gioia, 2004). Thereby, we aim to show how dynamic community interactions unfold among the identified concepts and to explain how online communities provide action possibilities for entrepreneurs that seek support.

To do so, we connected our emerging concepts on support seekers’ posts with subsequent community reactions and focused on the role of support providers and the action possibilities that they offered in the discussions (c.f. Gioia et al., 2012). Furthermore, we built a logical structure that shows how conversations of entrepreneurs develop in online communities by linking support seeker behavior to community reactions. Finally, we compared our model to various studies on affordances (e.g., Faraj & Azad, 2012; Leonardi, 2011, 2012, 2014, 2018; Leonardi & Treem, 2012) and refined concepts as well as relationships in our grounded theory model (c.f. Gioia et al., 2012). Thus, we made sure that the resulting grounded theory model on affordances of online communities in the entrepreneurship context (1) shows dynamic community interactions, (2) describes action possibilities that entrepreneurs can access in online communities, and (3) includes all relevant connections to affordance theory (c.f. Gioia et al., 2012).

## Results

The results of our grounded theory approach reveal a variety of both support-seeking behaviors of entrepreneurs as well as community reactions during the COVID-19 pandemic. Building on our analysis, we identify four specific affordances that emerge for entrepreneurs through their interaction with online communities: *resolving*, *reframing*, *reflecting*, and *refocusing*. In the following, we introduce each of the affordances.

### Resolving entrepreneurs’ specific problems

The first affordance that derives from our analysis is *resolving*, which means that entrepreneurs and the community collectively solve specific issues that arise due to the COVID-19 pandemic. Resolving unfolds through limited interaction between community and support seeker beyond an initial specific question, yet supports entrepreneurs in overcoming knowledge gaps, gaining tangible resources, and finding peer-sourced knowledge quickly (Fig. 1).

Thus, resolving as an action possibility arises when entrepreneurs seek *simple solutions without further interaction with the community.* Specifically, we identified three different support-seeking behaviors in our data: (1) entrepreneurs bring up specific operational problems that occurred due to governmental regulations, such as having to leave co-working spaces, missing pitch opportunities, coping with running costs, or applying to government loan programs. Operational problems are generally formulated clearly and concisely, asking the community for particular pieces of information or completing quick tasks, such as product tests. Specific operational problems represent a large variety of issues. For example, one founder faces difficulties opening a banking account because “IRS shut down all of these services no fax response no phone answering no mail processing” and asks whether the community has “any clue of alternative plans to obtain access to corporate banking.” Another founder is “looking for some really enthusiastic people to help us test and feedback the almost finalized version.” (2) Entrepreneurs ask for resources that help them to be more resilient during the COVID-19 crisis. These conversations usually start with an initial post that already suggests a few exemplary resources that help to cope with the COVID-19 pandemic. However, the general intention of the support seeker is that the community adds more resources to a list. For example, some support seekers create contact lists to help people find a new job, and others ask for online tools that are useful when working from home: “I’m here to make you a simple question: What are the most useful tools you know and use (…) for collaborative work ideation?” (3) Entrepreneurs look for resources to spend their extended leisure meaningfully. In contrast to entrepreneurs who ask for business-related resources, entrepreneurs who seek leisure resources do not collect previous thoughts or suggest an exemplary resource. These support seekers often explain their life situations and provide insights into why they are looking for books, podcasts, or films. For example, during quarantine, one entrepreneur looks for inspiration to start a business: “For this quarantine, I want to read as many books as I can to help me with starting up my own business (…) Which books under 200 pages would you recommend?” Taken together, entrepreneurs ask specific, narrow questions that address a need for precise information, particular task execution or online resources.

Resolving unfolds as the community responds to such initial posts by *closing knowledge gaps and providing tangible support* in addressing specific operational problems or a lack of resources during the COVID-19 pandemic. (1) The community provides informational support through precise answers that close knowledge gaps. For example, an entrepreneur asked for the amount of money that a company can get when participating in the government loan program of the USA and received the following answer: “10 k advance REGARDLESS of if you are approved or not (…) it's given on a first come first serve basis.” There is almost no interaction in these conversations, and they are regularly terminated when the knowledge gap is closed. For example, one entrepreneur is looking for a specific chair for his/her home office and receives the following answer: “The brand appears to be hon. [link]” In response to this, the support seeker left the community: “That’s it. Thank you. Stay safe.” (2) The community jointly creates resource lists of, e.g., online tools, books, or podcasts. For example, resources are collected in the comments: “Webex is offering full services on their free remote meeting accounts for 3 months.” Other community members create an openly accessible list: “If you’re compiling a list, can you throw it in a google doc?” (3) The community executes specific tasks, such as product tests, visiting or developing a website if requested by the support seeker. In these conversations, community members respond that they completed the task and talk about their experiences and impressions. For example, one community member tests a shop website and is wondering that “there’s not much actionable info.” To sum up, community members respond directly to support seekers’ specific problems and act to close knowledge gaps, jointly create resource repositories, and execute requested online tasks.

### Reframing entrepreneurs’ complex problems

The second affordance that we identify in our data is *reframing*. In case that support-seeking entrepreneurs ask nonspecific questions, the community challenges the initial post and reduces the complexity of the problem. Reframing unfolds when the community challenges assumptions and provides new perspectives on nonspecific questions (Fig. 1). Indeed, support-seeking entrepreneurs are not directly looking for new perspectives; they are rather unclear about what they want to get back from the community.

Reframing as an action possibility arises when problems are *not easy to solve because they involve high complexity and uncertainty*. Under those circumstances, entrepreneurs show three different support-seeking behaviors: (1) entrepreneurs ask nonspecific questions regarding operational problems and are unclear about what they want to get back from the community. For example, one entrepreneur runs a B2B startup and does not know how to acquire clients during the pandemic: “I was getting ready to launch a massive direct mail campaign to the execs at these companies right before the virus shut everything down. I’m not sure what to do now.” (2) Entrepreneurs seek for funding during the COVID-19 crisis as many companies are “facing a cash crunch” and funding sources are harder to access. However, instead of asking the numerous potential investors that are part of the community for direct funding, support seekers focus on information around funding opportunities and strategies. For example, a group of startup founders asks “At what stage is it okay to get an angel investor involved?” in place of directly turning to potential angel investors in the community. (3) Entrepreneurs look for feedback on ideas and strategy. Generally, these entrepreneurs do not know whether they should continue working on their project or abandon it. For example, one support seeker asks if community members think that “it’s a good idea to ride the wave” during the pandemic and to “open beta with a different vertical like essential goods.” Overall, we classify requests as reframing whenever entrepreneurs ask nonspecific questions, address broad topics, and request feedback on their ideas and strategy.

Reframing unfolds as the *community challenges assumptions and provides new perspectives* on a topic. (1) The community questions whether the information provided by the support seeker is correct and asks for clarification. Often, community members insist that the initial poster provides more details on the issue when missing information hinders support provision. In this regard, one community member mentions*:* “Nobody is going to be able to give you good advice if you’re going to be this vague.” (2) The community divides problems into subproblems to reduce the complexity. This approach enables support seekers to deal with their problems more easily. For example, one community member states that a support seeker who seeks funding should first create a financial plan, set demonstrable milestones, and acquire clients. (3) The community develops a completely new solution approach by providing creative ideas. For example, one person wrote about aiming to launch a rebranding and sought advice on how to proceed. However, the community does not support this approach during the COVID-19 pandemic: “Your focus should be to sell right now no need for a rebranding in a crisis situation.” All in all, in reframing discussions, community members aim to change the way entrepreneurs are looking at their problems.

### Reflecting entrepreneurs’ situations

The third affordance that is prevalent in our data is *reflecting*. Reflecting unfolds when community members and support seekers collectively engage in interactive discussions. Thereby, the community creates a common understanding of a topic (Fig. 1)*.*

Reflecting arises as an action possibility when support seekers try to make sense of a situation “in the COVID-19 era” through exchange with the community. More precisely, we identified three different support-seeking behaviors: (1) entrepreneurs wonder about the general economic situation for entrepreneurship during the pandemic. For example, one support seeker asks the community: “With the COVID-19 and recession looming, is now a bad time to start?” Another entrepreneur says that he/she “was putting together websites for clients right as the coronavirus pandemic struck and the interest just fell off a cliff” and asks whether people cannot afford new websites anymore. In opposite to the previous affordances, the support seekers here are not interested in solving a problem, but rather in discussing broader implications of the COVID-19 pandemic. (2) Entrepreneurs wonder about the impact of the pandemic on their business and ask the community about their experiences during the COVID-19 crisis. For instance, a group of entrepreneurs seeks “to understand how sales have been impacted for other startups (…) during the covid outbreak” as their startup is “pretty new. So, it’s hard to gauge mom (…) differences to really assess the damage this is doing.” (3) Entrepreneurs look for others that face the same challenges or have a similar mindset while coping with the pandemic. Interestingly, this support-seeking behavior is often driven by emotions. More precisely, these posts involve the need for compassion regarding life-changing decisions, as well as personal problems, and the wish to exchange with like-minded others who face similar challenges. For example, one entrepreneur states that “this is not the easiest time for humanity. There are a lot of crazy things happening and plenty of irresponsible people around (…) I somehow made a habit of looking out for the positive news aspects.” Further, the initial poster seeks people that share similar thoughts. Taking all three support-seeking behaviors together, entrepreneurs try to make sense of the pandemic in different ways.

Reflecting unfolds because the community responds to such an initial post by *creating a collective understanding of a topic*. (1) The community provides their shared assessment regarding the economic situation during the pandemic. For example, one community member states that he/she thinks that “there will be a large bounce. We’re going to have months of deferred purchases.” (2) The community provides information regarding the broader business impact of the COVID-19 crisis. For example, the community creates a collective understanding that due to the economic and social development during the pandemic, the idea of a support seeker “won’t work because most are cutting back on their expenses to prepare for the coming recession.” (3) The community explains best practices in several experience-discussion loops. For example, as many entrepreneurs have difficulties implementing online business-to-business marketing, a community member agreed that LinkedIn may support client acquisition during the pandemic. (4) The community collects ideas and opinions around a large variety of topics. For example, we identified a few discussions around work from home that do not directly relate to a problem but discuss opinions on the topic: “I’ve been in a remote job for a little over a year. Honestly if I had the choice I wouldn’t work from home (…) I’ve found much of the human element is stripped away in remote work” (5) The community shows compassion regarding fateful strokes and provides emotional support to overcome mental ill-being. For example, one founder explains that he is probably infected and that he needs to put himself in quarantine. One community member reacts as following: “Sorry you are going through this. My family and I are social distancing (as well).” To sum up, the community exchanges thoughts on various topics which develops into heated discussions and enables support seekers’ deeper reflection.

### Refocusing entrepreneurs’ thinking and efforts

The fourth affordance of *refocusing entrepreneurs’ thinking and efforts* enables entrepreneurs to open up new future actions and gain new options through community support. Our analysis shows that *refocusing* enabled entrepreneurs to broaden their future actions and “helped (…) connect a few more dots” so that they could “figure out next steps.” More precisely, the community interacts with support seekers to create forward-looking plans (Fig. 1).

Refocusing as an action possibility occurs when entrepreneurs *aim to plan future actions through strong interactions with the community*. In particular, we identified two types of support-seeking behavior: (1) entrepreneurs address complex strategic problems that emerged during the pandemic whereby they seek to define specific steps that go beyond reflection. For example, one co-founder asks for future steps to find a co-founder during the pandemic: “Given COVID-19 (…) most of my team has disbanded and the most passionate co-founders are leaving (…) it will be really hard to find someone who’s passionate enough to join me” (2) Entrepreneurs look for encouragement to undertake next steps whereby these support seekers especially ask for emotional support that can shape their way of thinking. For example, one support seeker wants to know how others “deal with growing pains and the mental exhaustion from having to scale at lightning speed” due to the COVID-19 pandemic.

Refocusing unfolds as the community responds to such requests *by interacting with support seekers to create forward-looking plans*. (1) The community makes suggestions for specific action-oriented plans. For example, one entrepreneur wants to know “the best way to find a technical partner in this quarantine world.” Then, a community member suggests: “You can check out the typical places developers hang out: google, some developer forums and groups… and join and preset some basics of your idea and what you intend to achieve.” (2) The community engages in personal exchanges with support seekers and often provides companionship, which means that support seeker and community members plan future actions together. For example, one support seeker is “looking for enthusiastic people to create something meaningful during the quarantine but seems like everyone is watching Netflix or playing video games.” Consequently, many community members want to get in direct contact to create a social business, i.e., make future plans, together with the support seeker: “I’m a Webdev and would be interested in collaborating. I’d like to do something productive during the quarantine” “I’m down! PM me.” (3) The community seeks to inspire support seekers to undertake future actions. Our findings indicate that in some cases, support seekers’ self-esteem increased through positive affirmation from the community, providing entrepreneurs with more confidence to undertake specific next steps and create future action plans. For example, one entrepreneur explains that his/her family does not support entrepreneurial activities and that they feel distracted in home office. Consequently, the entrepreneur asks: “How do I maintain my composure and focus on my projects? How do I stay resilient without driving myself crazy?” One community member responds: “Don’t let other opinions get to you. They are your family. Obviously, they want the safest thing for you. But you have to remember—entrepreneurs don’t like being safe and secure. We like to live in a life with no regret and making our own decision.” Taken together, community interactions potentially shape future actions and, therefore, can unfold in refocusing efforts and thinking.

### A theoretical model on affordances for entrepreneurs in online communities

**Fig. 2:**
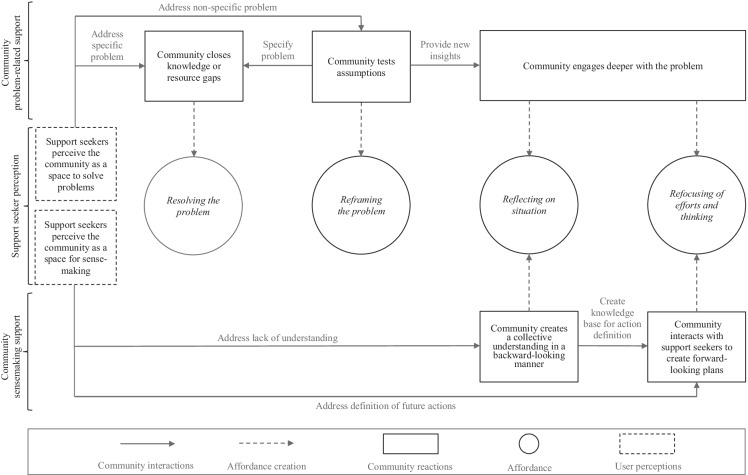
Affordances of online communities in the entrepreneurship context

Building on our findings, we develop a model on how the support-seeking behavior of entrepreneurs in online communities and community interactions create four affordances. The model outlines the following three steps: (1) support seeker perceptions, namely the goal expressions of the user interacting with technology (e.g., Faraj & Azad, 2012; Leonardi, 2017; Nambisan et al., 2019), (2) community reactions, thus the immediate community response to the behavior of the support seeker or other users (e.g., Joinson & Dietz-Uhler, 2002; Pfeil, 2009; Treem & Leonardi, 2013), (3) emerging affordances from the interactions between social agency (support-seeking entrepreneur) and the material agency (online community) (e.g., Autio et al., 2018; Kuo et al., 2013; Treem & Leonardi, 2013). Figure 2 shows how these steps are organized and highlights that depending on how support seekers perceive the community, namely whether they perceive it as a space to solve problems (upper part of Fig. 2) or space for sensemaking (lower part of Fig. 2), interactions differ, which can lead to varying community responses and further create action possibilities for entrepreneurs.

When support seekers perceive the community as a space for problem-solving, they either ask precise questions that they need an answer for or they describe nonspecific problems for which the solution path is unclear. Precise questions are usually related to specific, narrow problems that the community can solve by providing information and lists of resources. However, the form of engagement is limited by the lack of agency of the support seekers; they rarely respond or engage further with the community once their problem is solved, leading to limited interactions. Thus, the *resolving* affordance represents a case where the social agency of support seekers may limit the material agency of online communities (Leonardi, 2012). Interestingly, whereas the literature normally highlights deep and continued imbrications of human agency and material agency as the foundation of affordances (e.g., Leonardi, 2011), we see that limited interactions between actors (support seekers) and technology (online community) also can produce affordances, although they may be limited in nature. Indeed, while specific questions may limit the support potential of an online community, addressing complex problems can lead to community support that either provides the opportunity to simplify the initial submissions or provides new insights through challenging the original post. By simplifying a problem, the community reframes a nonspecific into a specific problem which further enables community members to directly tackle it through knowledge gap-filling or resource provision. In contrast, when community members challenge initial posts’ correctness or claim missing information, the *reframing* affordance provides support seekers with the opportunity to rethink their assumptions. Connecting this finding to the knowledge flow perspective of Faraj et al. (2016), our findings indicate that knowledge does not necessarily flow according to support seekers’ initial goals but might be redirected through new insights in online communities. Redirection of knowledge flows might, therefore, also lead to new community dynamics that cause community members to engage more deeply with the problem which consequently enables profound conversations.

However, when support-seeking entrepreneurs perceive the online community not just as a knowledge or resource repository (c.f. Kuhn et al., 2016, 2017) but as a space for sensemaking, entrepreneurs can access more fruitful discussions and thus draw on the affordances *reflecting on a situation* and *refocusing efforts and thinking*. In case that the support seeker mainly focuses on a lack of understanding, engaged discussions unfold that collect knowledge pieces and emotional impressions to create an overall understanding of a situation. Our findings indicate that these reflecting discussions can go beyond the already known affordances of knowledge sharing, knowledge source identification as well as knowledge creation (Faraj et al., 2011; Leonardi, 2014, 2018). While Leonardi (2014, 2018) rather highlights visibility and storage of knowledge, the reflecting affordance that we outline shows how emotions and information are combined to create a more complete understanding of topics. However, some support seekers do not aim to reflect on present or past situations but to define actions within community discussions in a forward-looking manner. Thereby, community and support seekers closely work together to jointly plan support seekers’ actions. More precisely, community and support seeker engage in several response feedback loops that ultimately help to adjust future actions according to the support seeker situation and needs. Out of this interaction, an affordance unfolds that can shape entrepreneurial action, especially during crises (c.f. Shepherd, 2020). Indeed, online communities can also stimulate entrepreneurial action in case that discussion shifts from a situation- and backward-oriented perspective into action- and future-oriented perspective.

To sum up, the model shows that depending on how support seekers perceive the online community, different affordances emerge. When entrepreneurs perceive it as space for problem-solving, online communities mainly provide the action possibilities to resolve problems or reframe problems. Indeed, due to community dynamics, these digital spaces can also enable reflection on situations and refocusing of efforts and thinking which was, however, not directly triggered by the support seeker. Furthermore, when entrepreneurs perceive online communities as spaces for sensemaking, they interact strongly with support seekers and thus offer a reflection on situations and refocusing regarding future actions.

## Discussion

Our findings and model provide new insights into *how* entrepreneurs exchange support in online communities during the COVID-19 pandemic. By relying on affordances as a theoretical perspective (e.g., Faraj & Azad, 2012; Leonardi & Vaast, 2017), we highlight how entrepreneurs gain access to four affordances through interactions with the online community. Building on these insights, we make two contributions.

### COVID-19, entrepreneurial action, and online communities

Many entrepreneurs are hit hard by COVID-19, both economically (Block et al., 2021; Cowling et al., 2020; Graeber et al., 2021) and emotionally (Giones et al., 2020). Entrepreneurs are, therefore, likely to need support to conduct entrepreneurial action (Kuckertz et al., 2020). Yet, there is a lack of knowledge from *where* entrepreneurs get the support to create the desired entrepreneurial action (Shepherd, 2020), especially when traditional, offline networks are not accessible (Majchrzak & Shepherd, 2021).

We show that online communities can serve as alternative sources where entrepreneurs can access crucial social support. While current research highlights especially the function of online communities as a knowledge repository (c.f. Kuhn et al., 2016, 2017), our findings indicate a broader set of social support that entrepreneurs may gain from online communities. *First*, entrepreneurial online communities function as spaces where users jointly collect crisis resources that are openly accessible and can help overcome COVID-19-related business issues (Kuckertz et al., 2020). Online communities provide entrepreneurs with a wealth of resources that they can immediately use (c.f. Hwang et al., 2015), which is especially important taking into account that fast responses to crisis are critical for the survival of new ventures (e.g., Giones et al., 2020; Korber & McNaughton, 2018). *Second*, online communities provide support that may reduce uncertainty after an exogenous shock. More precisely, drawing on the reframing affordance, the community challenges initial posts’ informational quality which means, for example, that venture launch plans undergo a feasibility assessment (c.f. McMullen & Shepherd, 2006) so that entrepreneurs can better evaluate opportunities (Autio et al., 2013). Furthermore, we found that online communities can create a more complete understanding of topics by combining information and emotion in deep reflections. In doing so, entrepreneurs were able to get a better understanding of work from home as this topic does not only create operational challenges but may also risk the mental health of employees and supervisors (c.f. Kniffin et al., 2020). *Third*, online communities can provide tailor-made plans for entrepreneurs by engaging in frequent interaction with the support seeker. Entrepreneurial action planning is particularly useful in times of high uncertainty (Giones et al., 2020) and may have a positive effect on survival (Delmar & Shane, 2003; Song et al., 2019) as well as performance (Chrisman et al., 2005) which makes online communities a critical infrastructure.

Naturally, as an inductive study on a very recent phenomenon, our claims to all three effects are tentative. Yet, our claims do find support in recent studies that echo similar sentiments (Giones et al., 2020; Kuckertz et al., 2020; Kuhn et al., 2017). Hence, it is plausible that online communities could be a missing link in understanding how entrepreneurs can obtain crucial support in times of crisis, which facilitates their entrepreneurial action. For example, when an unexpected crisis, such as a fire or earthquake, hits ( Shepherd & Williams, 2020; Williams & Shepherd, 2016), the entrepreneur’s local community may not possess the knowledge to alleviate the situation and help the entrepreneur. Yet, it is plausible that such knowledge exists in other places, which may be accessible through online communities. Consequently, we encourage future research to investigate the role of online communities in providing crucial support that facilitates entrepreneurial action, not just in the context of COVID-19, but in other crises as well, where entrepreneurial action may be crucial (Korsgaard et al., 2020; Shepherd & Williams, 2020). More precisely, drawing on our findings, we encourage future research to investigate (1) the role of online communities in assembling crisis resources, (2) online community mechanisms that reduce uncertainty, and (3) the impact of online community support on business planning in times of crises.

### Entrepreneurship and digital affordances

We outline four digital affordances that entrepreneurs can draw upon in online communities. Thereby, we contribute to the recent interest in how digitalization affects entrepreneurship and how entrepreneurs can make use of digital affordances (Autio et al., 2018; Nambisan, 2017; Sahut et al., 2019). Our findings outline that online communities are “malleable” and can offer different things depending on how entrepreneurs perceive online communities and make use of them.

Depending on their perception of the online community, entrepreneurs receive different responses from the community and unpack different affordances of the community. Here, our study enlightens recent debates in network research concerning entrepreneurs’ networking style (Hallen et al., 2020; Vissa & Bhagavatula, 2012). The networking style originally referred to whether entrepreneurs sought to broaden or deepen their networks (Vissa, 2012), but recently Hallen and colleagues (Hallen et al., 2020) suggested that scholars pay more focus on entrepreneurs’ agentic actions, which refers to how entrepreneurs grasp resources and support from their networks. To this recent theorization, we add insight into how entrepreneurs gain value from online communities, and in particular, how different ways of perceiving and approaching online communities result in different affordances. In other words, we unpack how the perception of entrepreneurs matters greatly for what they gain from using online communities. A particular feature of our data is that we can capture the activity and interactions of entrepreneurs, something which common quantitative research tools in network research do not allow for. Thus, we pose that future researchers interested in the networking style of entrepreneurs could rely on social media data, such as online community data but also other sources, such as emails (c.f. Saxton & Wang, 2014). This may open for a fine-grained understanding of how entrepreneurs actively use their networks.

Furthermore, we draw out how affordances differ not just in the perception of entrepreneurs but also the level of community engagement (Faraj & Azad, 2012; Krogh et al., 2012). Our model highlights the importance of deep community engagement in enabling affordances of reflecting and refocusing. Indeed, support seekers that perceive digital spaces as means for solving problems may also limit the support potential of online communities through a lack of engagement. Furthermore, when entrepreneurs give online communities a higher meaning, i.e., when they focus on sensemaking, discussions can provide support beyond simple knowledge flows (Faraj et al., 2016) by combining emotions and information to create a more complete understanding of issues and enable future actions and orientations of entrepreneurs (c.f. Autio et al., 2013). Moreover, we show and explain how online communities may simultaneously act to reduce and increase the complexity of topics, shifting an information-related discussion toward a more emotional and reflective dimension. As such, our findings indicate the interrelatedness of digital affordances and provide impetus for further study of this interplay.

*Finally*, our findings can also provide a foundation for future research into digital affordances in other areas. For example, research has argued that online communities play a crucial role in mobilizing crowdfunding (Nambisan, 2017). Yet, if online communities are malleable and offer different affordances depending on entrepreneurial perception and action, it is important to analyze online communities as fluid tools that are dependent on how entrepreneurs engage with them. In other words, the affordances that entrepreneurs can access in online communities with regard to crowdfunding may be diverse and context-dependent (c.f. Oo et al., 2019; Piezunka & Dahlander, 2019; Skirnevskiy et al., 2017). Following the affordance perspective, it is not the technology itself that provides affordances, it is the interaction between people and technology (Faraj & Azad, 2012). The affordance perspective has gained some foothold in recent conceptual papers on the intersection between entrepreneurship and digitalization (Autio et al., 2018; Nambisan, 2017), but to our knowledge, we present the first empirical study that unpacks digital affordances that entrepreneurs can access. Thus, our study can provide a foundation for future research to empirically investigate digital affordances, which is useful given the agreement that the affordance perspective is a crucial tool for entrepreneurship scholars to unpack the impact of digitalization on entrepreneurs (Nambisan, 2017; Sahut et al., 2019).

### Practical implications

Beyond theoretical contributions, our findings provide actionable implications for entrepreneurs by uncovering under which conditions entrepreneurs are likely to receive support and which opportunities they have participating in online communities. Depending on the initial framing and interaction with the community, we show how support seekers can influence the support they receive from online communities. We, therefore, recommend entrepreneurs to fit their support-seeking behavior toward the goal they want to reach. More precisely, we advise support seekers with specific problems to formulate and contextualize their problems and specify what kind of information or resource they need. Furthermore, we suggest entrepreneurs who want to reduce the complexity of an issue to highlight uncertainty about the framing of a problem. Also, we recommend generalizing problems for more community engagement that enables a deeper reflection about a situation. Moreover, we advise entrepreneurs to get strongly involved in discussions if they want to generate future action possibilities. Finally, entrepreneurs mainly looking for emotional support should be willing to open up to the community so that counterparts understand their need for compassion and empowerment.

## Limitations and conclusion

As an inductive study on an ongoing and novel phenomenon, our paper is not without limitations. *First*, our data only covers one online community, which, although very large with over 460,000 members in July 2020, may limit the transferability of our findings to other online communities. We thus urge future research to investigate and compare different online communities. *Second*, while we focus on the current COVID-19 crisis, we do not know how generalizable knowledge from this crisis is. For example, we cannot yet observe if our findings are generalizable to other crises dealt with in the entrepreneurship literature, such as earthquakes (Shepherd & Williams, 2020). *Third*, while our findings draw out important processes of support exchange, we do not have data that covers focal outcomes, i.e., we do not know whether the support that entrepreneurs receive translates into increased venture creation, further entrepreneurial growth, or improves entrepreneurs’ mental health. Due to these limitations, we encourage future research to test whether the use of online communities has a positive impact on venture creation and entrepreneurs’ mental health.

In sum, this article investigates how entrepreneurs utilize online communities during COVID-19 to seek support, drawing out how interactions between entrepreneurs and the community provide four unique affordances. Our study thereby contributes to our knowledge about how entrepreneurs deal with the COVID-19 crisis, how online communities function as social support networks in comparison to traditional physical networks, and how entrepreneurs access digital affordances. Altogether, we hope that our study can serve as an inspiration for further research at the intersection of entrepreneurship and digitalization. Echoing recent research (Nambisan, 2017; Nambisan et al., 2019), we believe there is much to gain studying entrepreneurship in digital spaces, both in times of crisis and in normal times.

## Figures and Tables

**Fig. 3:**
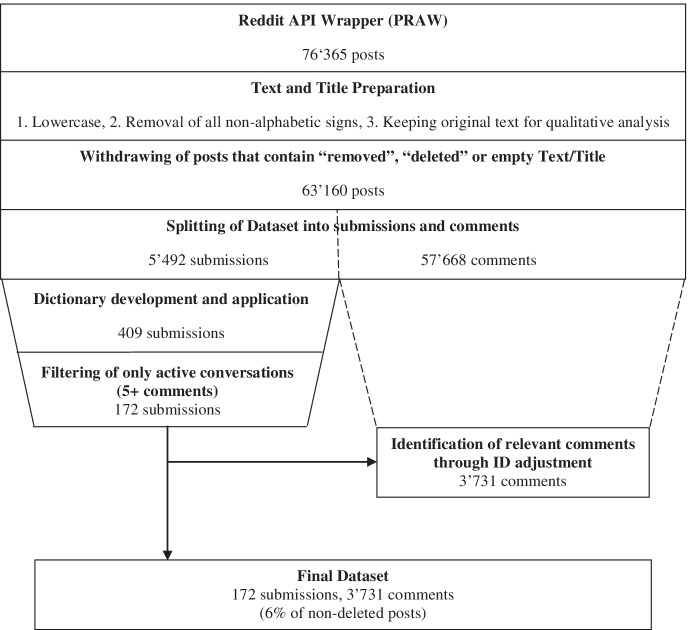
Data filtering process

**Table 2:**
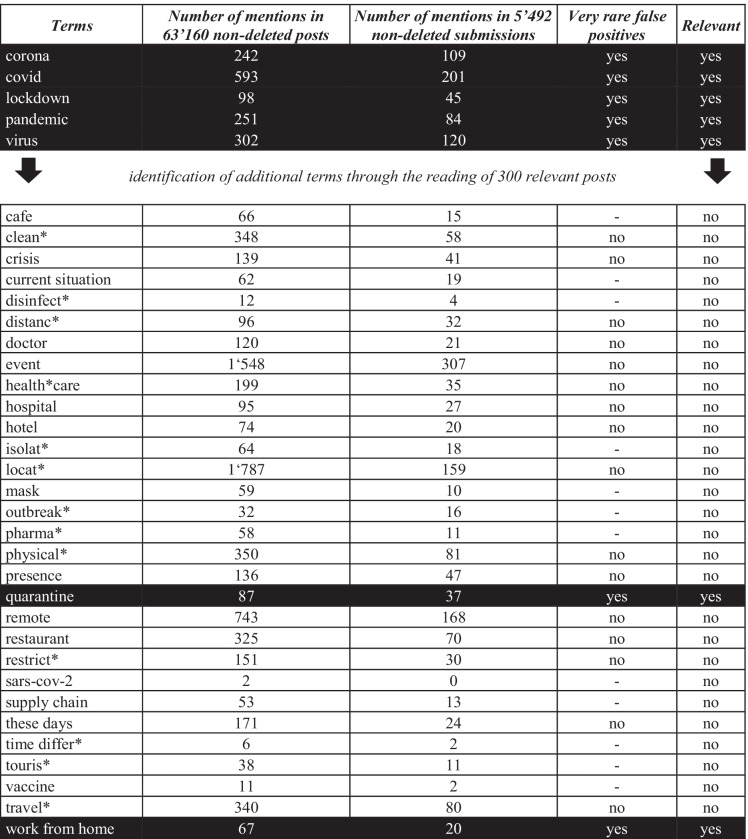
COVID-19 dictionary; *Relevant* =  > *50 Mentions Total,* =  > *20 Mentions in Submissions,* < *5% of the submissions include false positives.*

## Data Availability

The dataset in this study was scraped from an online community of entrepreneurs on Reddit (r/startups) by using a self-developed script within the Python Pushshift.io API Wrapper**.** The data that support the findings of this study is available from the corresponding author, Marie Madeleine Meurer, upon reasonable request.

## Appendix 2: Additional insights into the dataset (Table 3)

**Table 3:** Codes and representative quotes

**Codes**	**Representative quotes**
***Resolving***	
***Support seekers look for simple solutions without further interaction with the community***	
*Support seekers address specific operational problems related to the COVID-19 pandemic*	*“My mum has been tasked with finding EU rules and regulations on how companies and especially AI can handle patient’s personal data. She is finding it really hard because she doesn’t know where to look for this info.”* *“We’re looking for some really enthusiastic people to help us test and feedback the almost finalized version.”* *“How do I formulate a proper pitch presentation?”* *“I’m just looking for help on defining who I’ll be transferring these funds to at the end of the day. I want the people to be able to have as much choice as possible in where their funds go because every city will experience different problems in this crisis.”* *“Since we are quarantined due to covid and it might be a while, I would love to order that chair for myself and use it in my home office. Does anyone know the make and brand of chair used in WeWork?”*
Support seekers ask to collect (free) resources that help to be more resilient during the COVID-19 pandemic	*“Have you guys switched to work from home culture to continue your businesses and just to minimize the losses and to fight the recession phase in this global pandemic? If you have faced problems doing so with respect to marketing and customer support, so kindly list them.”* *“I am building a network of health professionals offering pro bono advice for the covid *via* video conference. Can anyone point me to solutions that are robust cheap and don’t require from patients to download an app? Browser-based the obvious ones are Google Hangouts, Meet, or Skype. But I was wondering whether a solution closer to telehealth exists.”* *“Google and Microsoft recently made a switch to make enterprise free to help out people are any other companies doing the same thing feel free to share them here.”* *“To help unemployed Canadian startup workers amid covid you can add your profile here to be seen publicly by employers.”* *“What are the most useful tools you know and use as a startup free or not for collaborative work; ideation, innovation frameworks, brainstorming, design thinking, *etc*. Now with the quarantine, I need some really good online tools to digitalise most of the daily work.”*
Support seekers look for resources to spend their extended leisure meaningfully during the pandemic	*“Here are ivy league courses you can take online right now for free (…) If you have anything more please share in the comments.”* *“I would like to try to read as much as possible before I go back to work.”* *“What innovation resources are available websites, books, mentors (during the pandemic).”*
***Community closes knowledge or resource gaps***	
Community provides short, clear answers to problems	*“I just emailed the local EU office and they helped me for free with detailed answers!”* *“You must have US bank accounts. You can use a transferwise account without an EIN and still send receive USD.”* *Support seeker is looking for a particular chair; “The brand appears to be hon.”* *“Sorry, I wasn’t clear I meant you make a personal account. In the meanwhile, if you absolutely need banking right now and can’t wait the several weeks for the IRS. It’s a temporary solution.”*
Community creates a resource base	*“You need to take care of cloud collaboration tools. For that, we use g-suite, Gmail, drive, hangouts, meet, gatlabs.”* *“Worth a reread of Ben Horowitz classic”* *“Quick reselling eCommerce platform offering free set up of eCommerce stores during the covid pandemic. It is a good opportunity for small businesses and startups also the company has waived off transaction charges in this pandemic”* *“This is a good resource to help you out with writing your first pitch: [link]”* *“If you are looking to set up meetings and talk to people through collaboration then you might consider cisco, Webex, Microsoft teams, slack, ring central, and nine (…) those are a few of the collab tools, I have worked with before and they have their own features and interfaces that you can use.”*
Community executes requested tasks such as product tests or website development	*“It is simple. Do not worry, I can add some input!”* *“That’s so sick. I love stuff like that. I wish I had more time to tinker. I’m possibly looking for a part-time salesperson in Canada SaaS company. So, I’ll look through that list.”* *“Todd upvoted and followed you.”*
***Reframing***	
***Support seekers ask non-specific questions***	
Support seekers look for funding during the pandemic	*“Since I obviously did not plan for a pandemic to be happening, and since no one knows how long it will go on for, I have no idea what to do. I do have the funds to keep the servers going until November without any income but after that, I’ll have to close up shop.”* *“Sadly, I have no capital but I’m willing to take out loans to support this idea. But due to the cost of app development, I alone will not be able to raise sufficient funds. So I’d be looking for an angel investor.”* *“With the covid situation, revenue is down as the startup relates to events. That is not the biggest deal as I see this downtime.”* *“Hey, I am interested to hear what other founders, VCs experts think about fundraising in a recession. We are closing a seed round and our founding team are speculating.”* *“We’re a group of three people. We are currently facing some cash crunch due to the ongoing pandemic and have been looking forward to get some investment by giving equity.”*
Support seekers ask non-specific questions regarding operational problems	*“I was getting ready to launch a massive direct mail campaign to the execs at these companies right before the virus shut everything down. I’m not sure what to do now.”* *“I was wondering if anyone has any tips (…) if any is involved in making the post a success. What we’ve built is a really robust platform with great UX that we are super proud of and I want to showcase it in the best possible light.”* *“How long is it taking to incorporate a c-corp in Delaware now because of coronavirus and is it still possible to get the EIN, no? Which would be the best company to use in these times for the incorporation?”* *“As covid worsens in the EU whereas we can see some recovery in Asia what would be the best thing the community here would do? Should we send a few of our top-performing employees over to Asia to push for BD?”* *“The team is lacking a lead developer. The pandemic only makes the situation worse at least in my city where people are all in lockdown state and so I’m considering hiring a freelance developer for the time.”*
Support seekers look for feedback on ideas or strategy	*“Not entirely sure what to do with them but want to use them to help provide (…) testing location info.”* *“I thought folks might be interested in what kind of traffic and conversion numbers we saw. I’m guessing these are atypical considering that we’re in the middle of a pandemic.”* *“I was thinking about exporting goods to African countries where they lack basic necessities. My idea is to arrange goods from across different countries.”* *“I dunno if it's something people are even searching for. What do you think? Feedback appreciated [link]. Any ideas on how to explain present the concept to people?”* *” We’re considering going open beta with a different vertical like essential goods e.g. hand soaps, gloves, *etc*. Question: Think it's a good idea to ride the wave or hold out and hope the category recovers? Curious if others are considering pivoting things right now.”*
***Community tests assumptions***	
Community questions post information correctness or ask for clarification regarding missing information	*“What's the need to rebrand when the sale is happening?”* *“What do you mean protect yourselves? What are you protecting yourselves from?”* *“Nobody is going to be able to give you good advice if you're going to be this vague.”* *“When the outcome is putting your other employee’s lives at risk, I feel like this is not the appropriate way to look at things.”* *“Agreed. The wording is super confusing. Have you found any clarity?”*
Community divides problems into subproblems and explains how to deal with these subproblems	*“I mean saying you want to discount the rent can be writing up an addendum to your contract. If they accept, you are good. I’m not sure what other options you have besides organizing a tenants union and negotiate based on many business in the same circumstances.”* *“I usually define my problem and search how others did it. When I learn a new step or process I do a deep dive until I understand it, then try and solve my problem, then google the error codes.”* *“You should be achieving demonstrable milestones at least every three months whether technical or customer; not clear on whether in time sales or recurring revenue recurring is much more valuable.”* *“Take your cash flow with all the milestones you may have up until you become cash flow positive. (…) A good of contingencies is the amount you will need equity call if the amount is too high you’ll have to split it in different rounds lay them on a timetable and understand exactly what you are going to achieve at each milestone. Keep your burn rate waaaay under the limit that will bridge you to the next funding because excel files are not real life if you need a dcf template or a cap table with a funders dilution roadmap.”* *“If you had a family therapist for example with patience, you could in theory have a beta test group of users instantly, instead of trying to get them one at a time, because it is being offered in an environment where people are already paying for a service.”*
Community identifies alternative solution approaches	*“Your focus should be to sell right now. No need for a rebranding in a crisis situation!”* *“Is there a reason you can’t go out to a coffee shop or other public environment a few times a week?”* *“Well, think about who’s affected by this problem and interview them. You don’t have to intentionally lead the convo towards the problem but ask them about their lives and how they do things currently are doing things in this area.”* *“Is there way you could modify your business plan or offer a product that could be useful during a lockdown? It’s here for a few months.”* *“I’d say it’s less about recruiting and more just finding someone with the same goals as you.”*
***Reflecting***	
***Support seekers try to make sense of the situation through information exchange***	
Support seekers wonder about the general economic situation during the pandemic	*“The startups that’ll shape the future this time will be different although depressing. This recession could have many profound benefits from reducing the harm on the environment to improving the way we communicate and work. The coronavirus means more people will work from home so we’re seeing a rise in the use of remote working tools already people are reporting on the improved quality of life from the temporary changes in their work habits. This is all pretty obvious. What else are we missing? What positive changes, new startups, and exciting opportunities do you think will come out of this uncertain period?”* *“With the covid and recession looming, is now a bad time to start? It seems like people are in full panic mode and probably trying to save as much money as possible.”* *“Covid has seen the economy shaken quite a bit with many businesses bearing the brunt.”* *“I was putting together websites for clients right as the coronavirus pandemic struck and the interest just fell off a cliff. Not good. Maybe I’m wrong but I think people still want to build stuff, they just can’t afford it right now.”* *“I am interested to hear what other founders VCs experts think about fundraising in a recession.”*
Support seekers wonder about the impact of the pandemic on their individual business	*“Does anybody here consider applying for this small business support program?”* *“Are you noticing a significant slow down in engagements (…)? If you are seeing this as well I assume it’s primarily that everyone's organic feeds are filled up with covid based information and non-virus stuff is getting buried.”* *“Those of you with B2B startups are doing to land deals right now? I was getting ready to launch a massive direct mail campaign to the execs at these companies right before the virus shut everything down. I’m not sure what to do now.”* *“Just a small startup I’ve been working on for three years blood sweat tears and all of that our whole country is on lockdown and last week we exploded I’ve never had to maneuver and make so many adjustments to our operations in such a short period of time.”* *“I’m trying to understand how sales have been impacted for other startups so far during the covid outbreak and how they are forecasting sales the rest of the year.”*
Support seekers look for others that face the same challenges or have a similar mindset while coping with the pandemic	*“I think now is an interesting time to talk about mental health in industry and the startup world should we expect an incoming urgency to track and manage employee wellness perhaps a surge in hr tech what is happening has been happening at your startup in terms of managing wellbeing.”* *“Have you guys switched to work from home culture to continue your businesses and just to minimize the losses and to fight the recession phase in this global pandemic?”* *“This is not the easiest time for humanity. There are a lot of crazy things happening and plenty of irresponsible people around. However amidst all of this chaos, many positive things are showing through: People are singing in balconies during quarantines, various businesses and people donating resources to hospitals.”* *“I’m looking for enthusiastic people to create something meaningful during the quarantine but seems like everyone is watching Netflix or playing video games.”* *“Ok, so I’ve decided to use this coronavirus time to my advantage and launch a project I’ve always wanted to do.”*
***Community creates a collective understanding in a backward-looking manner and through deep engagement with a problem***	
Community provides information regarding the economic situation during the pandemic	*“The car bubble finance is expected to be the one that pops first will be interesting to see where that places your offering.”* *“I think there will be a large bounce. We’re going to have months of deferred purchases, lots of employees are getting govt monies.”* *“I’m not so sure people lost their jobs or worked less and will have to pay the frozen bills all at once.”* *“I wouldn’t be surprised if trump dropped taxes for the rest of the year for an additional economic kick.”* *“I think throwaway restaurant is referencing the stock market circuit breakers were due to such a sharp drop trading is halted to try to restore restive trading stability. I could be wrong though.”*
Community provides information regarding business impact of the COVID-19 crisis	*“I wouldn’t just ignore this. The earlier you prepare the better off your business will be in handling it. If you don’t, you may wake up one day to find half your staff out sick with no contingency plan.”* *“If it actually works, it’s a great time. If all you are doing is offering marketing, it won’t because most are cutting back on their expenses to prepare for the coming recession.”* *“You could start a hand sanitizer business you’d be rich within a month.”* *“Early Feb was a time when many places still weren’t in lockdown. I’m an engineer and direct marketer and I know for a fact that business owners that have nothing to do with covid are actually spending less on their advertising right now(…). Perhaps you should test your advertising again. What niche is it for and what type of advertising have you tried?”* *“I don’t think this is an opportunity to make money. Of course, we’re being negative. The world economy is being temporarily shut down that’s the reality. My take: use this time to plan out what your next moves might be, once we do come out of this difficult period. People are going to be craving some things they couldn’t do for a while. Jump on that wagon and you’ll make bank but right now is not a time to thrive it is a time to survive.”*
Community explains best practices (during the COVID-19 pandemic)	*“The top comments here present a couple of reasonable options holding till things change or negotiating a contract that accommodates you if you keep working without being paid.”* *“Pick one (freelancer) in your own country as that helps communication and if you can find one in your own timezone even better.”* *“Put yourself on LinkedIn and state exactly what you wrote here in the summary section. Be bold in your ask. Don’t pretend to know everything and be willing to do the grunt work to learn go for it and read everything you can.”* *“Facebook is the ultimate example of a product going viral. I too am confused by what the person above you is saying within hours of launching the site had active users. He obviously wasn’t spending a penny on marketing at this point.”*
Community collects subjective impressions around a large variety of topics	*“I’ve been in a remote job for a little over a year. Honestly, if I had the choice I wouldn’t work from home all of the time. I’ve found much of the human element is stripped away in remote work never realized.”* *“I personally do not support this trend as gambling has even permeated gaming in the form of loot boxes.”* *“Having an army definitely helps but it’s not always necessary. We made it to the top of the day without me even realizing someone else posted us. Just like any marketing channel the success of your launch is a function of both your product and distribution. It’s almost never binary.”* *“There is a huge difference between; do what you love with a calculated plan and just try and hope for the best. I’m not saying he shouldn’t do it because of failure or fear but he should have a plan and a backup plan.”* *“That’s not true at all. I’ve been an entrepreneur for years with about different entities across LLCs and c2s corps. My wife is an accountant and startup advisor reimbursing owner and employee expenses is an absolutely normal process for a business.”*
Community shows compassion regarding fateful strokes	*“It sounds like you’re taking some sensible steps to help yourself. Well done. Remember to congratulate yourself for every victory you make on your way to recovery.”* *“Sorry you are going through this, my family and I are social distancing for my son’s spring break. We will see if we can last in each other’s constant company for days.”* *“Try to remain civil in how you communicate with others. Even if you feel they are being extremely foolish, I know that can be extremely difficult but rule is important to us, and crossing that line will be met with appropriate action.”* *“Dude, that’s awesome man. I’m so happy to hear that’s going for you. I don’t know why the mod is talking smack. It’s a good thing to share this. So, people can get certain ideas. Congrats man, the only thing that counts is if you can keep up the pace.”* *“I’m sorry I made you feel disturbed with my fear of being replaceable.”*
***Refocusing***	
***Support seekers aim to define future actions through strong interactions with the community***	
Support seekers address complex strategic problems that emerged during the pandemic	*“My company is now segmenting employees based on different locations and packs it diversifies risk. But I still don’t think it’s the best idea as everyone commutes from all around *etc*. But it still poses risk thanks and would appreciate how some startups are doing this so we can get an idea too.”* *“I just started making a line of handbags and I realized it’s really expensive to produce inventory. I was curious where I could find funding and what other routes I could take to fund my new business. I was going to produce in china but because of the shipping costs and coronavirus I think I rather produce in the US or Mexico.”* *“I sold my shares and made a significant amount with crypto that I can reinvest in a startup I would start. I’ve recently been wanting to go back into the startup world but not too sure how to go at it.”* *“It will be really hard to find someone who’s passionate enough to join me. He stated that it’s a limiting belief that I can’t find someone even if I don’t have an idea. Apparently, if you’re a good recruiter you can persuade someone to do something bigger than themselves.”* *“What stage in the business I would need to be at to make this a viable investment? How to make sure I’m not ripped off and my idea isn’t stolen?”*
Support seekers look for encouragement to undertake next steps	*“We have no reason to be in the office other than giving each other this false sense of work getting done. I might take a hike voluntarily because I have at-risk people at home who’s survival I would very much like.”* *“We became a very essential tool due to covid. We had to scale insanely fast and therefore had to cut corners. So, last week when our user base went up by, we crashed several times. It felt like the end of the world. I had anxiety attacks all week long.”* *“This is a wild time and I feel very fortunate I’ve been giving back to the community in various ways but I don’t want to look like I’m patting myself on the back (…) does anyone else have a company that unexpectedly started thriving because of the virus? What are you doing about it? Are any of you with companies able to help in your communities at this time? If so, what are you doing?”* *“I have a business idea that’s a niche version of an existing website. I’d like to pitch the idea to the original site’s CEO.”*
***Community interacts with support seekers to create forward-looking plans through deeper engagement with a problem***	
Community makes suggestions for future actions	*“Find a cool coworker space and camp there during your work hours. Socializing is great in such places if you have the correct attitude.”* *“Could you find a mentor, an advisor you trust already to work with you and help you revise your sales funnels and scripts, someone that would work for equity or revenue share or straight commission?”* *“Don’t take a loan it’s too naive to think that no one has ever thought of a similar idea. Find the target users, show them your idea and see if they hand you over their wallet.”* *“No point in getting stressed out, customer can squeeze if you let them. You have to be diplomatic where ever required in service. You are right in terms of challenges. You focus on product and it’s very important to keep yourself not stressed out because no one else. It’s a fight.”* *“I’d try writing to give valuable information and build awareness and tie it to a relevant offer they could benefit from immediately. I mean if your product helps people, why limit the help they can get from you? Offer them more value for a good bargain price.”*
Community engages in personal contact with support seekers and often provide companionship	*“Guys pm me I have got fews projects where I get juniors who want to collaborate in great startups.”* *“I can offer help on the front end. Can you explain what you’re working on?”* *“Can you pm me the link? Would love to check out if it’s not a problem.”* *“Send me dm if you need help with that. I specialize in digital media and paid social advertising and I’m a big art fan.”* *“Happy to brainstorm together and share my experience on how I’ve found others to brainstorm with. I’m in NYC with a background in healthcare will dm you resume current project contact info.”*
Community seeks to inspire support seekers to undertake future actions	*“Congrats on the MVP and being in a decent spot. It sounds like you’ll have some time to find good technical leadership without your hair on fire or at least more on fire than usual.”* *“He said he’s in high school. LOL, what kind of work can he possibly have done? He seems passionate, why don t you give him a chance.”* *“That’s incredible, it takes a lot just to keep the lights on. Great work on pivoting so quickly.”* *“You can go for it—decision is purely yours. If joining summer offers means short term loss but long term gain in terms of knowledge and skills, then you can consider going for the offer.”* *“That sounds awesome. Sorry for misunderstanding the business model. I’d say, ask for more you’ll need to have tons of runway. I hope to be a customer soon.”*

## Code availability

The code for scraping the Reddit community data is available from the corresponding author, Marie Madeleine Meurer, upon reasonable request.
